# Quartet-based inference is statistically consistent under the unified duplication-loss-coalescence model

**DOI:** 10.1093/bioinformatics/btab414

**Published:** 2021-05-28

**Authors:** Alexey Markin, Oliver Eulenstein

**Affiliations:** Virus and Prion Research Unit, National Animal Disease Center, USDA-ARS, Ames, IA 50010, USA; Department of Computer Science, Iowa State University, Ames, IA 50011, USA

## Abstract

**Motivation:**

The classic multispecies coalescent (MSC) model provides the means for theoretical justification of incomplete lineage sorting-aware species tree inference methods. This has motivated an extensive body of work on phylogenetic methods that are statistically consistent under MSC. One such particularly popular method is ASTRAL, a quartet-based species tree inference method. Novel studies suggest that ASTRAL also performs well when given multi-locus gene trees in simulation studies. Further, Legried *et al.* recently demonstrated that ASTRAL is statistically consistent under the gene duplication and loss model (GDL). GDL is prevalent in evolutionary histories and is the first core process in the powerful duplication-loss-coalescence evolutionary model (DLCoal) by Rasmussen and Kellis.

**Results:**

In this work, we prove that ASTRAL is statistically consistent under the general DLCoal model. Therefore, our result supports the empirical evidence from the simulation-based studies. More broadly, we prove that the quartet-based inference approach is statistically consistent under DLCoal.

**Supplementary information:**

Supplementary data are available at *Bioinformatics* online.

## 1 Introduction

The accurate inference of evolutionary histories of species is a grand challenge in evolutionary biology due to the fact that the *true* evolutionary histories are rarely known (Bininda-Emonds, 2004). Consequently, the common strategy in the phylogenetic community is to rely on established statistical models of evolution when evaluating phylogenetic inference methods. One of the most prominent such models is the multispecies coalescent model (Rannala and Yang, 2003) that accounts for incomplete lineage sorting (ILS), also known as deep coalescence. ILS is a prevalent factor that causes discordance between the observed gene tree topologies and the host species tree (Allman *et al.*, 2018). In fact, a large body of work in phylogenetics is dedicated to the design of species tree inference methods that are statistically consistent under MSC. Statistical consistency implies that as the number of observed gene trees grows, the species tree estimate converges to the true species tree that ‘generated’ the observed data. Multiple phylogenetic inference methods have been demonstrated to be statistically consistent, cf. GLASS (Mossel and Roch, 2010), R${}^{*}$ (Degnan *et al.*, 2009), STEM (Kubatko *et al.*, 2009), MP-EST (Liu *et al.*, 2010), BUCKy (Larget *et al.*, 2010), STAR/USTAR (Allman *et al.*, 2016; Liu *et al.*, 2009), NJst (Liu and Yu, 2011), ASTRID (Vachaspati and Warnow, 2015), ASTRAL (Zhang *et al.*, 2018), other rooted triplet and unrooted quartet methods (Ewing *et al.*, 2008; Rhodes, 2020; Yourdkhani and Rhodes, 2020) and others.

**Fig. 1.**
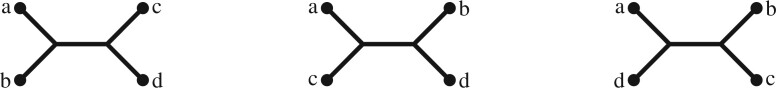
All three possible quartets on a,b,c,d leaves

In recent years ASTRAL became one of the most popular species tree inference methods by practitioners. Note that ASTRAL’s objective function is built on the notion of *quartets* (see Fig. 1). In particular, the proof that ASTRAL is statistically consistent under MSC stems from two observations. First, Allman *et al.* (2011) demonstrated that if a species tree displays a quartet *q* then *q* is also the most likely observed (unrooted) gene tree topology. Second, it can be seen that every species tree clade will eventually appear in at least one of the observed gene trees.

More recently, Legried *et al.* (2020) studied two natural extensions of ASTRAL that enable processing the multi-locus gene trees. Multi-locus gene trees can have multiple leaves with the same species label (that is, the respective species has multiple copies of the same gene). These extensions allow one to apply ASTRAL to a much broader class of phylogenetic gene trees and are referred to as *ASTRAL-one* and *ASTRAL-multi*. Given four species (e.g. {A,B,C,D}) a multi-locus gene tree can have multiple copies of each of the species and therefore can suggest multiple (conflicting) quartets on {A,B,C,D}. In that case, ASTRAL-one chooses a *single random* copy for each species label and considers the respective quartet type, whereas ASTRAL-multi considers *all* gene copies and all the respective quartets.

**Fig. 2.**
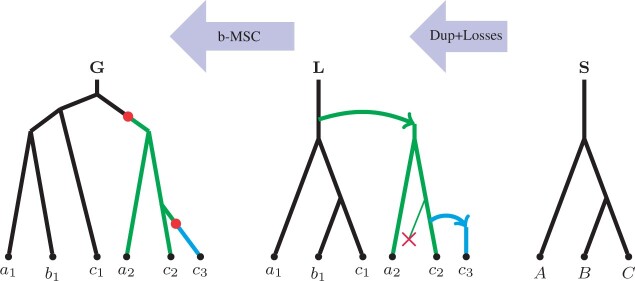
An example of a gene tree *G*, locus tree *L* and species tree *S*. Note that the arrows in the locus tree represent the duplication events, and the cross represents a loss event. Further, the red circles on the gene tree represent the duplication-points. As coalescent (b-MSC) runs on the locus tree, the coalescence of the new and the original loci is likely to happen above a duplication event; therefore, the duplication-points can appear in the middle of gene tree edges, as shown in the figure

Focusing on these two extensions of ASTRAL, Legried *et al.* proved that both ASTRAL-one and ASTRAL-multi are statistically consistent under the gene duplication and loss model (GDL) (Legried *et al.*, 2020). Note that GDL is a part of the broader and well-recognized unified duplication-loss-coalescence (DLCoal) model of gene tree evolution by Rasmussen and Kellis (Rasmussen and Kellis, 2012). DLCoal simultaneously accounts for three crucial types of evolutionary factors that shape gene family evolution. Namely, duplications, losses and incomplete lineage sorting. The DLCoal process involves two steps, (i) a birth/death process within the branches of the species tree creates a *locus tree* (i.e. the GDL process), and (ii) a bounded multispecies coalescence process acting on the locus tree generates the observed *gene tree*. See Figure 2 for an example.

In this work, for the first time, we prove that ASTRAL-one is statistically consistent under the general DLCoal model. First, we derive gene tree probabilities (constrained to quartets) under the bounded multispecies coalescent model and draw core observations from that analysis. Second, we build on an idea from Legried *et al.* to systematically separate different duplication-loss scenarios. Then, for each such scenario, we prove that a random quartet from the gene tree is more likely to agree with the species tree quartet rather than any of the two other quartets. Finally, we extend our result for ASTRAL-one to ASTRAL-multi and demonstrate that ASTRAL-multi is also consistent under DLCoal (Our extension of the consistency result to ASTRAL-multi was developed independently from Hill *et al.*, 2020.).

Our results provide a theoretical justification to the findings in Du *et al.* (2019), which showcased the accuracy of ASTRAL-one in the presence of duplications, losses and incomplete lineage sorting.

## 2 Preliminaries

We denote a rooted *(phylogenetic) tree* by P=(T,ω). Here *T* is the *tree topology* and is a binary rooted tree with the designated root vertex, ρ(T), of degree two, all internal nodes of degree three and with leaves bijectively labeled by elements of set Le(T). For convenience, we identify leaves with their labels. Further, tree topologies are *planted*, implying that an additional *root edge* is attached to the root vertex. Then, *ω* specifies the lengths of edges in *T* in *coalescent units* [i.e. the number of generations normalized by the effective population size (Allman *et al.*, 2011)]. More formally, $\omega :E(T)\to Q^{+}$. In particular, we assume that all edge lengths are strictly positive. When the phylogenetic tree *P* is not clear from the context, we will often use the notation *T_P_* and *w_P_* to refer to its tree topology and its edge-length function, respectively.

An *unrooted (phylogenetic) tree topology T* is similar to the rooted tree topology, but without a designated root and the root edge. That is, in unrooted tree *T* all non-leaf vertices have degree three.

We say that an edge *e* is *external* if it is incident with a leaf vertex, and otherwise we call *e internal*. Further, given a set Y⊂Le(T), tree topology $T{|}_{Y}$ is obtained from *T* by restricting the leaf-set to *Y*. A restricted phylogenetic tree $P{|}_{Y}=(T{|}_{y},w{|}_{Y})$ is then obtained by choosing the function $w{|}_{Y}$ that maintains the same leaf-to-root path lengths as in *P* (in respect to the leaves in *Y*).

A rooted topology *T* defines a partial order on its nodes: given two nodes *x* and *y* we say x ⪯ y if *x* is a descendant of *y* (and x≺y if additionally x≠y). We say that two edges in a rooted tree are *parallel* if neither edge is located on the path from the other edge to the root.

*Quartets*. A quartet is an unrooted tree topology with exactly four leaves. Assuming that the leaves are *a*, *b*, *c* and *d*, we denote the quartets in Figure 1(left), (middle) and (right) as ab|cd, ac|bd and ad|bc respectively (based on the two cherries separated by the internal edge).

We say that a quartet *q* is *displayed* in a phylogenetic tree *P*, if the unrooted tree topology of *P* restricted to the leaves in *q* (i.e. $T_{P}{|}_{\mathrm{Le}(q)}$) is equivalent to *q*. In this case, we write q∈P.

### 2.1 Unified DLCoal model

We now overview the unified duplication-loss-coalescence (DLCoal) model (Rasmussen and Kellis, 2012).

*Species tree*. A *species tree* $S=(T_{S},\omega_{S})$ represents an evolutionary history of species. Leaves of *T_S_* are labeled by the extant species names.

*Locus tree*. A *locus tree* $L=(T_{L},\omega_{L})$ represents a duplication/loss history of a fixed gene. A locus tree is obtained from a species tree by running the duplication/loss process (Legried *et al.*, 2020; Rasmussen and Kellis, 2012) top-down along the edges of the species tree. More specifically, the duplication/loss process is a birth-death process with a fixed birth (duplication) rate *λ* and death (loss) rate *μ* (Arvestad *et al.*, 2003). The birth-death process starts in the root edge of the species tree; whenever it reaches a speciation point, the process splits into two copies and continues independently in the children edges. See Figure 2 for an example. Note that locus tree leaves are labeled by gene names.

A locus tree node is always one of the following two types:

- *Speciation*. Such node corresponds to a speciation event/node from the species tree.
- *Duplication*. Such node corresponds to a new locus creation event.

**Fig. 3.**
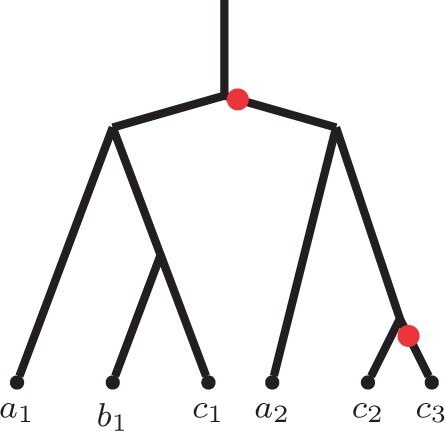
An alternative depiction of a locus tree from Figure 2 with red dots representing duplication-points *slightly* shifted toward a novel locus

Remark. *A duplication event is asymmetric, as one child (the mother duplicate) follows the parent locus, and the other child (the daughter duplicate) corresponds to a novel locus (Rasmussen and Kellis, 2012). To account for that, we will often depict duplications as red dots on the locus tree edges immediately below the duplication nodes, shifted toward a daughter duplicate. That is, a red dot on an edge will indicate that this point is a start of a new locus (see*  *Fig. 3*  *for an example). This will ensure a consistent depiction of duplications for* Section 3*. Further, we will refer to these points as duplication-points.*

*Gene tree*. A *gene tree* $G=(T_{G},\omega_{G})$ represents a gene family’s evolutionary history. The gene tree is obtained from a locus tree by running the *bounded multispecies coalescent (b-MSC)* process bottom-up along the edges of the locus tree (Rasmussen and Kellis, 2012) (see Section 2.3 for a more detailed description of that process). Figure 2 provides an example of that process.

### 2.2 Multispecies coalescent (MSC) model

In the standard multispecies coalescent model (Rannala and Yang, 2003) gene lineages are followed backwards in time (from the leaves to the root).

For simplicity, we assume that there is exactly one gene lineage starting in every extant locus tree leaf. If two or more lineages enter the same locus tree edge, then the coalescence history of these lineages is determined by an exponential distribution.

In particular, for any two lineages *a*, *b* that entered the same edge the probability that they coalesce within time *x* (specified in terms of coalescent units) is as follows:

$$P[a,b\;\text{coalesced}\;\text{within}\;\text{time}\;x]=1-e^{-x}.$$

More generally, we denote the probability that *i* lineages coalesce into *j* lineages within time *x* (j≤i) by $g_{i,j}(x).$ This value can be computed using the following formula (Tavaré, 1984):

$$\begin{array}{cc} g_{i,j}(x)=\sum_{k=j}^{i}( & \;\text{exp}\;\left(-\left(\begin{array}{c} k\\ 2 \end{array}\right)x\right)\frac{(2k-1){\left(-1\right)}^{k-j}}{j!(k-j)!(j+k-1)}\\ & \cdot \prod_{m=0}^{k-1}\frac{\left(j+m)(i-m\right)}{i+m}). \end{array}$$

Further, note that if at any given moment in time multiple lineages co-exist in the same edge, then any pair of these lineages have an equal probability of coalescing in the next Δt time. That is, the process is symmetric.

### 2.3 Bounded MSC (b-MSC) model

The constraints on MSC in the unified DLCoal model appear due to the duplication points. In particular, all lineages originating below a daughter duplicate must coalesce below the respective duplication node. For example, in Figure 3, the gene tree lineages corresponding to leaves *a*_2_, *c*_2_ and *c*_3_ must coalesce below the root node.

**Fig. 4.**
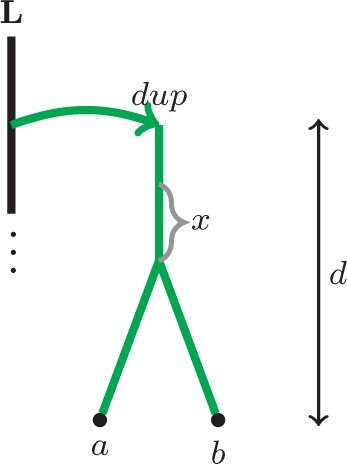
An example of a locus tree that illustrates the b-MSC constraints for Section 2.3

More formally, assume that a duplication occurred at time-point *d*. Note that, for convenience, we assume that all leaves are aligned in time and are associated with time-point 0; further, we consider time to increase as we go up the trees away from leaves. Now, let *a* and *b* be locus tree leaves that are located below the duplication, which is at time-point *d* (i.e. *a* and *b* belong to the new locus created by the duplication). Then we know that lineages *a* and *b* must coalesce prior to time-point *d*. Therefore, generally, the probability that any two lineages *a*, *b*, which entered the same edge below a duplication *dup* at time *d*, coalesce within time *x* is as follows (see Fig. 4 for a respective locus tree example):

$$\begin{array}{cc} & P[a,b\;\text{coalesced}\;\text{within}\;\text{time}\;x|a,b\;\text{coalesced}\;\text{prior}\;\text{to}\;\mathrm{dup}]\\ & \;=\frac{1-e^{-x}}{P[a,b\;\text{coalesced}\;\text{prior}\;\text{to}\;\mathrm{dup}]}, \end{array}$$

where P[a,b coalesced prior to dup] is determined by the original, unbounded MSC model.

## 3 Quartet probabilities under b-MSC

To obtain our main result we need to compute the probabilities of each quartet appearing in the gene tree based on a fixed locus tree topology. Note that Allman *et al.* (2011) explicitly computed these probabilities for unbounded MSC. In our case we need to incorporate cases, when duplications (locus creation events) appear along the edges of the locus tree.

Remark. *From now on, for convenience, we restrict locus trees to four leaves sampled from different species. That is, choosing (any) four genes* {a,b,c,d}  *from distinct species* A,B,C,D*, we consider the tree* $L{|}_{\left\{a,b,c,d\right\}}$*. Note that considering only four leaves may suppress other duplication nodes along the locus tree edges. Therefore, we need to allow for additional duplication-points along the locus tree edges. Further, if there are multiple duplication-points along a single edge of* $L{|}_{\left\{a,b,c,d\right\}}$*, it is sufficient to only consider the lowest duplication-point on that edge since it indicates the lowest point, below which gene lineages must coalesce.*

Without loss of generality assume that the locus tree *L* displays the quartet ab|cd. Then there are two cases: either (i) *L* is a balanced rooted tree or (ii) *L* is a caterpillar tree. We now explore both those cases.

Throughout this section, we sometimes use abbreviations ‘coal.’ for ‘coalesce(d)’ and ‘dup.’ for ‘duplication’. Further, we abbreviate ‘obtained in time *t*’ as simply ‘in *t*’.

### 3.1 *L* is balanced

**Fig. 5.**
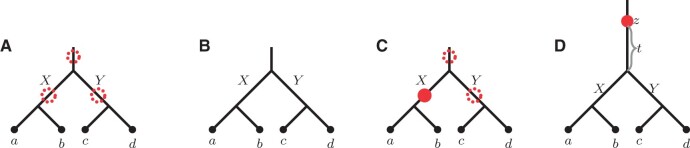
(**A**) The balanced quartet representing the locus tree and displaying quartet ab|cd. The dotted circles indicate potential duplication locations that can affect gene tree probabilities. (**B–D**) Specific duplication scenarios corresponding to Sections 3.1.1, 3.1.2 and 3.1.3, respectively

For convenience, we set $x:=\omega_{L}(X),y:=\omega_{L}(Y)$ to be the lengths of edges *X* and *Y*, respectively (see Fig. 5A). We now explore all possibilities of duplication placements on edges of *L*.

#### 3.1.1 No duplications (unbounded MSC)

In this case, quartet probabilities are given by Allman *et al.* (2011). That is,

$$\begin{array}{c} P[\mathrm{ab|cd}\in G]=1-\frac{2}{3}e^{-(x+y)};\\ P[\mathrm{ac|bd}\in G]=P[\mathrm{ad|bc}\in G]=\frac{1}{3}e^{-(x+y)}. \end{array}$$

#### 3.1.2 Duplications along the *X* or *Y* edges

Assume that a duplication has occurred along the *X* and/or *Y* edge (see Fig. 5C). Recall that a duplication point indicates that gene lineages below it in the locus tree must coalesce prior to the duplication (when looking backwards in time). Therefore, if there is a duplication along the *X* edge, then lineages corresponding to genes *a* and *b* must coalesce on that edge. That is, the gene tree must display quartet ab|cd. Similarly, the same is true if a duplication is located on the *Y* edge. Hence,

$$\begin{array}{c} P[\mathrm{ab|cd}\in G]=1;\\ P[\mathrm{ac|bd}\in G]=P[\mathrm{ad|bc}\in G]=0. \end{array}$$

#### 3.1.3 Root edge duplications

Assume that a duplication occurred on the root edge as shown in Figure 5(D), and no duplications appear on *X* and *Y* edges. Then the following holds.

$$\begin{array}{cc} & P[\mathrm{ab|cd}\in G]=P[\mathrm{ab|cd}\in G|a,b,c,d\;\text{coalesced}\;\text{before}\;z]\\ & =1-P\left[\begin{array}{cc} & a,b\;\;\text{did}\;\text{not}\;\text{coal}.\;\;\text{on}\;\;X;\;\;\\ & c,d\;\;\text{did}\;\text{not}\;\text{coal}.\;\;\text{on}\;\;Y;\\ & \mathrm{ac|bd}\;\text{or}\;\mathrm{ad|bc}\;\text{in}\;t\;\; \end{array}|a,b,c,d\;\;\text{coal}.\;\;\text{before}\;\;z\right]\\ & =1-\frac{\frac{2}{3}e^{-x}e^{-y}P[4\;\text{lineages}\;\text{coalesced}\;\text{within}\;\text{time}\;t]}{P[a,b,c,d\;\text{coalesced}\;\text{before}\;z]}\\ & =1-\frac{2}{3}e^{-(x+y)}\frac{g_{4,1}(t)}{P[a,b,c,d\;\text{coalesced}\;\text{before}\;z]}; \end{array}$$

 

$$\begin{array}{cc} P[\mathrm{ac|bd}\in G] & =P[\mathrm{ad|bc}\in G]\\ & =\frac{1}{3}e^{-(x+y)}\frac{g_{4,1}(t)}{P[a,b,c,d\;\text{coalesced}\;\text{before}\;z]}. \end{array}$$

#### 3.1.4 Duplication at the root vertex

In Sections 4 and 5 we mainly consider cases when the locus tree root corresponds to a locus creation event (i.e. there is a duplication-point on one of the *X* or *Y* edges right below the root). In that case the gene tree quartet probabilities are given by Lemma 3.1.

Lemma 3.1. *Let L be a balanced locus tree displaying a quartet q with the root of L corresponding to a duplication. Then* P[q∈G|L]=1.

Proof. Since the root of *L* is a duplication, we place a duplication-point immediately below the root on one of the children edges (i.e. the edge that corresponds to a novel locus). Therefore, quartet probabilities for *L* are described in Section 3.1.2. That is, P[q∈G|L]=1. □

Remark. *Note that potential duplications along the external edges do not affect the coalescence process.*

### 3.2 *L* is a caterpillar

**Fig. 6.**
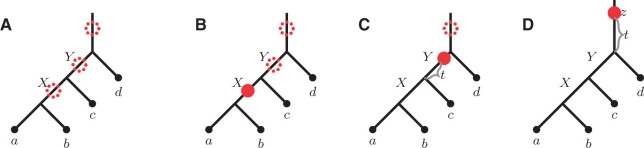
(**A**) The caterpillar quartet representing the locus tree and displaying quartet ab|cd. (**B–D**) Specific duplication scenarios corresponding to Sections 3.2.2, 3.2.3 and 3.2.4, respectively

As above, we set $x:=\omega_{L}(X),y:=\omega_{L}(Y)$ to be the lengths of edges *X* and *Y* respectively (see Fig. 6A). We now similarly explore all possible duplication placements on the edges of *L*.

#### 3.2.1 No duplications (unbounded MSC)

In this case, the quartet probabilities are given by Allman *et al.* (2011). In particular,

$$\begin{array}{c} P[\mathrm{ab|cd}\in G]=1-\frac{2}{3}e^{-x};\\ P[\mathrm{ac|bd}\in G]=P[\mathrm{ad|bc}\in G]=\frac{1}{3}e^{-x}. \end{array}$$

#### 3.2.2 *X* edge duplication

Assume that there is a duplication on the *X* edge (and potentially more duplications on other internal edges) as shown in Figure 6B. Then, similarly to the balanced case, it is not difficult to see that

$$\begin{array}{c} P[\mathrm{ab|cd}\in G]=1;\\ P[\mathrm{ac|bd}\in G]=P[\mathrm{ad|bc}\in G]=0. \end{array}$$

#### 3.2.3 *Y* edge duplication

Assume that there is a duplication on *Y* and there are no duplications on *X* as shown in Figure 6C.

$$\begin{array}{c} \begin{array}{cc} & P[\mathrm{ab|cd}\in G]=P[\mathrm{ab|cd}\in G|a,b,c\;\;\text{coal}.\;\;\text{before}\;\;\text{duplication}]\\ & =1-P\left[\begin{array}{cc} & a,b\;\text{did}\;\text{not}\;\text{coalesce}\;\text{on}\;X;\;\;\\ & \mathrm{ac|bd}\;\text{or}\;\mathrm{ad|bc}\;\text{in}\;t\;\; \end{array}|a,b,c\;\;\text{coalesced}\;\;\text{before}\;\text{dup}.\right]\\ & =1-\frac{2}{3}e^{-x}\frac{g_{3,1}(t)}{P[a,b,c\;\text{coalesced}\;\text{before}\;\text{duplication}]}; \end{array}\\ P[\mathrm{ac|bd}\in G]=P[\mathrm{ad|bc}\in G]=\frac{1}{3}e^{-x}\frac{g_{3,1}(t)}{P[a,b,c\;\;\text{coal}.\;\;\text{before}\;\text{dup}.]}. \end{array}$$

#### 3.2.4 Root edge duplication

Assume that a duplication occurred on the root edge and no duplications occurred along the *X* and *Y* edges (see Fig. 6D).

We start with computing the probability of the ac|bd quartet.

$$\begin{array}{c} \begin{array}{cc} P[\mathrm{ac|bd}\in G] & =\frac{P\left[\begin{array}{cc} & a,b\;\text{did}\;\text{not}\;\text{coalesce}\;\text{on}\;X;\\ & a,c\;\text{coalesced}\;\text{on}\;Y\;\text{first};\\ & \text{remaining}\;\text{lineages}\;\text{coalesced}\;\text{before}\;z \end{array}\right]}{P[a,b,c,d\;\text{coalesced}\;\text{before}\;\text{the}\;\text{duplication}]}\\ & +\frac{P\left[\begin{array}{cc} & a,b\;\text{did}\;\text{not}\;\text{coalesce}\;\text{on}\;X;\\ & \text{no}\;\text{coalescence}\;\text{on}\;Y;\\ & \mathrm{ac|bd}\;\text{obtained}\;\text{in}\;\text{time}\;t \end{array}\right]}{P[a,b,c,d\;\text{coalesced}\;\text{before}\;\text{the}\;\text{duplication}]} \end{array}\\ \;=\frac{\frac{1}{3}e^{-x}(g_{3,2}(y)g_{3,1}(t)+g_{3,1}(y)g_{2,1}(t)+g_{3,3}(y)g_{4,1}(t))}{P[a,b,c,d\;\text{coalesced}\;\text{before}\;\text{the}\;\text{duplication}]}. \end{array}$$

Further, by symmetry P[ac|bd∈G]=P[ad|bc∈G]. Therefore, P[ab|cd∈G] equals

$$1-\frac{2}{3}e^{-x}\left(\frac{g_{3,2}(y)g_{3,1}(t)+g_{3,1}(y)g_{2,1}(t)+g_{3,3}(y)g_{4,1}(t)}{P[a,b,c,d\;\text{coalesced}\;\text{before}\;\text{the}\;\text{duplication}]}\right).$$

### 3.3 Core observations

It is not difficult to see from the above derivations that for a fixed locus tree topology that displays ab|cd (balanced or caterpillar), if one increases the length of edge *X* then the probability P[ab|cd∈G] grows. More formally, see Lemma 3.2.

**Fig. 7.**
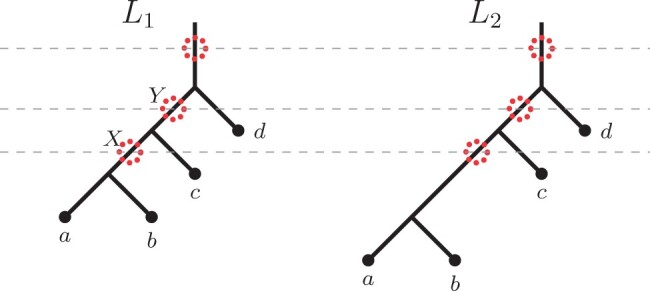
Locus trees *L*_1_ and *L*_2_ with equal lengths of the *Y* edges and different lengths of the *X* edges. The dashed lines highlight that the duplication-points are located identically on the two trees relatively to their roots

Lemma 3.2. *Let L_1_ and L_2_ be two caterpillar trees displaying* ab|cd  *with* $\omega_{L_{1}}(X)<\omega_{L_{2}}(X)$  *and* $\omega_{L_{1}}(Y)=\omega_{L_{2}}(Y)$  *as shown in Figure 7. Further, let L_1_ and L_2_ have identical locations of duplication-points on the internal edges. That is, a duplication-point d_1_ on L_1_ always has a counterpart duplication-point d_2_ on L_2_ with the same distance to the root and vice versa (see Fig. 7). Then*
 

$$P[\mathrm{ab|cd}\in G|L_{1}]=P[\mathrm{ab|cd}\in G|L_{2}]=1,$$

*if L_1_ (and L_2_) have a duplication-point on edge X, and*
 

$$P[\mathrm{ab|cd}\in G|L_{1}]<P[\mathrm{ab|cd}\in G|L_{2}],$$

*otherwise.*

Further, from the above derivations we observe the following lemma.

Lemma 3.3. *For a locus tree L that displays* ab|cd  *(regardless of duplication locations) we have* P[ab|cd∈G|L]>P[ac|bd∈G|L]=P[ad|bc∈G|L].

The proofs of Lemmas 3.2 and 3.3 are given in Supplementary  Section S1 of Supplementary Material.

## 4 Consistency of ASTRAL-one

We now prove ASTRAL-one is statistically consistent under the DLCoal model.

Theorem 4.1. *Let* $S=(T_{S},w_{S})$  *be a fixed species tree and let* G  *be a collection of gene trees that independently evolved within S according to the DLCoal process. Then, as the number of trees in* G  *goes to infinity, the probability that* $\hat{T}$*, the unrooted tree estimate by ASTRAL-one, is equal to the unrooted tree topology T_S_ goes to 1.*

For this result, it is sufficient (see Legried *et al.*, 2020) to prove the following:

Theorem 4.2. *Let S be a species tree with four leaves that displays quartet* AB|CD*, and let G be a gene tree that evolved in S according to the DLCoal process. If one picks genes* a,b,c,d  *(that correspond to species* A,B,C,D  *respectively) uniformly at random (assuming they exist) from G, then* P[ab|cd∈G]>P[ac|bd∈G]=P[ad|bc∈G].

Theorem 4.2 is sufficient to prove Theorem 4.1, because ASTRAL, as a distance-minimization method, ‘prefers’ the most dominant quartets among the input trees. Then, by Theorem 4.2, as the number of input trees goes to infinity, the most dominant quartet among input trees for each 4-tuple of species becomes (*almost surely*) the true species tree quartet; hence, it is almost surely picked by ASTRAL-one (see Legried *et al.*, 2020 for a formal proof). Therefore, the remainder of the section is dedicated to the proof of Theorem 4.2. We first prove the theorem for *S* being balanced and then for *S* being a caterpillar.

Remark. *To prove Theorem 4.2, we will use some of the results from Legried et al. (2020), who proved that ASTRAL is consistent under the duplication/loss process. To see how their result relates to our problem, observe that a ‘gene tree’ in Legried et al. (2020) notation is equivalent to the locus tree in the broader DLCoal process. Therefore, below we explicitly use some of Legried et al. results to draw conclusions about the locus tree probabilities.*

### 4.1 *S* is balanced

**Fig. 8.**
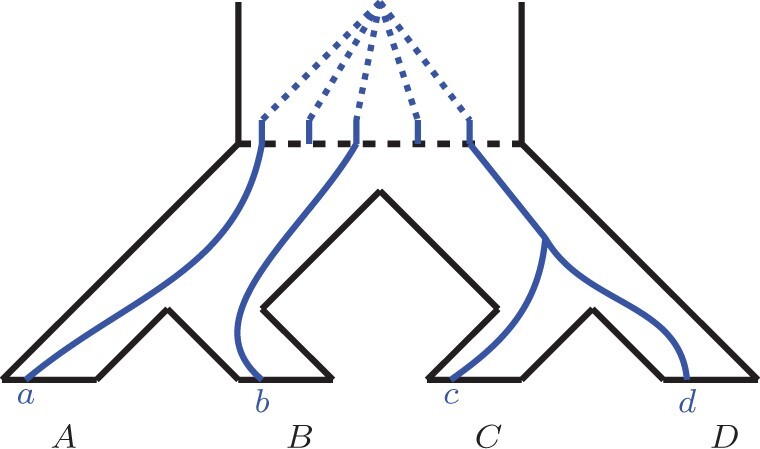
An example of the partial embedding of a locus tree into balanced *S*. The blue lineages correspond to the locus tree. Note that the five locus lineages crossing the dashed speciation line are *root lineages*

Similarly to Legried *et al.* (2020), we first implicitly condition our probability space on the event that at least one of each *a*, *b*, *c* and *d* genes must be present in *G*. Further, we condition our probability space on a fixed number of locus tree lineages existing at the speciation point at the root of *S*. That is, consider the duplication/loss (birth/death) process occurring within the root branch of *S*. Then, let *RL* be the random variable denoting the number of locus lineages at the speciation point (see Fig. 8). We are going to prove that

$$\begin{array}{cc} P[\mathrm{ab|cd}\in G|RL=l] & >P[\mathrm{ac|bd}\in G|RL=l]\\ & =P[\mathrm{ad|bc}\in G|RL=l] \end{array}$$

for any fixed value of l={1,2,…}. Therefore, for convenience, we do not explicitly write the condition *RL* = *l* in probability equations throughout the rest of the proof. Further, we refer to the set of these *l* locus lineages as *root lineages*.

Now let $i_{a}\in \{1,\ldots ,l\}$ be the index of a root lineage, from which gene *a* has descended. Similarly, we define *i_b_*, *i_c_* and *i_d_*. For better readability of the remainder of the proof, we introduce the notation to describe scenarios of the type $i_{a}=i_{b}=i_{c}\neq i_{d}$. In particular, we write (*abc*, *d*) for that scenario, we write (*ab*, *cd*) to denote the scenario $i_{a}=i_{b}\neq i_{c}=i_{d}$, and we write (a,b,c,d) to denote the scenario, where all *i_x_* are distinct.

Then, by the law of total probability, we have

$$P[\mathrm{ab|cd}\in G]=\sum_{I}P[I,\mathrm{ab|cd}\in G],$$

where *I* is one of the above scenarios (i.e. a partition of set {a,b,c,d} or a combination of such partitions). In particular I∈{(a,b,c,d);(ab,cd)∨(ac,bd);(ab,c,d)∨(cd,a,b)∨(ac,b,d)∨(bd,a,c);(abc,d)∨(abd,c)∨(acd,b)∨(bcd,a)∨(abcd);(ad,bc)∨(ad,b,c)∨(bc,a,d)}. Observe that we cover all possible scenarios/partitions here.

Our goal is to prove that P[ab|cd∈G]>P[ac|bd∈G]. Note that P[ad|bc∈G]=P[ac|bd∈G] follows from the fact that swapping *c* and *d* leaf labels does not affect the probabilities. Let us carry out the proof by considering different values of *I*. That is, our strategy is to prove that P[I,ab|cd∈G]≥P[I,ac|bd∈G] for all of the above *I*, and at least in one case the *strict* inequality holds.

To facilitate the proofs in each case, first consider the following observations:

Observation 4.1. *Random variables i_x_ and i_y_ are independent for any* x∈{a,b}  *and* y∈{c,d}.

*However, i_a_ can be dependent on i_b_ and i_c_ can be dependent on i_d_.*

Proof. Observe that the duplication/loss process runs independently in the parallel branches of the species tree. Therefore, once we condition the probability space on a fixed number of lineages at the divergence point (i.e. fixed *l*), the random variables *i_x_* and *i_y_* become independent. In particular, consider any specific realization of the duplication/loss process below the root lineages and let *i* be a root lineage that a randomly picked locus *a* belongs two (i.e. *i_a_* = *i*). Then, we can swap the ‘left’ subtrees below two distinct root lineages *i* and *j* (the subtrees that lead to species *A* and *B*) so that *i_a_* = *j* and the probability of that event is not altered due to symmetry. Note that *i_c_* in that case remains the same. Since we can always reshuffle root lineages like that, we can think of *a* as ‘choosing’ one of the *l* root lineages uniformly at random, regardless of a realization of *i_c_*. The same is also true for all other pairs of x∈{a,b} and y∈{c,d}.

However, since *a* and *b* develop (at least partially) in the same species tree branch random variables *i_a_* and *i_b_* can be dependent. Similarly for *i_c_* and *i_d_*. □

Observation 4.2. *Due to the symmetry of the duplication/loss process, we have*
 

$$P[i_{x}=k]=1/l$$

*for any* x∈{a,b,c,d}  *and* k∈{1,2,…,l}*. Then, by Claim 4.1*,

$$P[i_{x}=i_{y}]=\sum_{k=1}^{l}P[i_{x}=k]P[i_{y}=k]=l\frac{1}{l^{2}}=1/l$$

*for any* x∈{a,b}  *and* y∈{c,d}.

Lemma 4.1 (Due to Lemma 1 in Legried *et al.*, 2020). $P[i_{a}=i_{b}]$ and $P[i_{c}=i_{d}]$ are greater than or equal to $\frac{1}{l}$.

#### 4.1.1 Case *I = (a,b,c,d)*

By the symmetry of the duplication/loss process, reshuffling the $i_{a},i_{b},i_{c},$ and *i_d_* labels will not change the probability of a fixed duplication/loss history in the root edge. Therefore, we have P[ab|cd∈G|I]=P[ac|bd∈G|I]. Hence, P[ab|cd∈G,I]=P[ac|bd∈G,I].

#### 4.1.2 Case I=(ab,cd)∨(ac,bd)

We need to show that

$$\begin{array}{cc} P[\mathrm{ab|cd}\in G,I] & =P[\mathrm{ab|cd}\in G|(ab,cd)]\;P[(ab,cd)]\\ & \;+P[\mathrm{ab|cd}\in G|(ac,bd)]\;P[(ac,bd)]\\ & \geq P[\mathrm{ac|bd}\in G|(ab,cd)]\;P[(ab,cd)]\\ & \;+P[\mathrm{ac|bd}\in G|(ac,bd)]\;P[(ac,bd)]\\ & =P[\mathrm{ac|bd}\in G,I]. \end{array}$$

Observe the following.

Lemma 4.2. P[ab|cd∈G|(ab,cd)]=P[ac|bd∈G|(ac,bd)]=1.

**Fig. 9.**
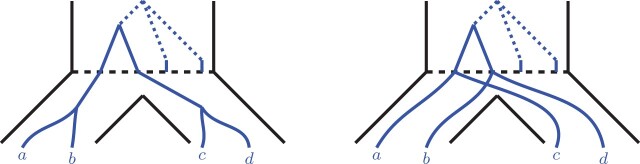
Left: the embedding of a locus tree $L_{\left(ab,cd\right)}$. Right: the embedding of a locus tree $L_{\left(ac,bd\right)}$

Proof. Consider the locus trees $L_{\left(ab,cd\right)}$ and $L_{\left(ac,bd\right)}$ for the (*ab*, *cd*) and (*ac*, *bd*) cases respectively (see Fig. 9). Note that we only consider the part of the locus tree restricted to the four selected genes a,b,c,d. It is not difficult to see that both $L_{\left(ab,cd\right)}$ and $L_{\left(ac,bd\right)}$ are balanced. Therefore, by Lemma 3.1, P[ab|cd∈G|(ab,cd)]=P[ac|bd∈G|(ac,bd)]=1. □

Corollary 4.1. P[ac|bd∈G|(ab,cd)]=P[ab|cd∈G|(ac,bd)]=0.

Lemma 4.3.
P[(ab,cd)]≥P[(ac,bd)] .

**Fig. 10.**
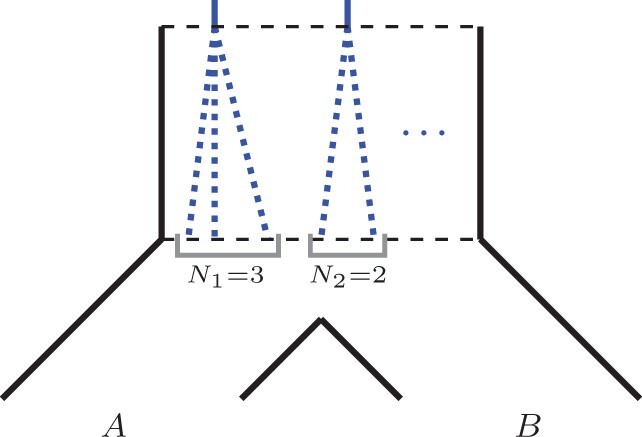
An example of a partial locus tree embedding in the left part of the species tree below the root speciation. The two shown root lineages expand (through duplication) into $N_{1}=3$ and $N_{2}=2$ lineages at the moment of *A*/*B* speciation, respectively

Proof. Our proof is similar to the proof of Lemma 1 in Legried *et al.* (2020). In particular, let $N_{i}\in \{0,1,\ldots \}$ be the number of locus lineages that descended from a root lineage i∈{1,…,l} and that existed *immediately after* the speciation into species *A* and *B*. Similarly, we define variables *M_i_* denoting the number of lineages that existed immediately after the speciation at the parent of *C* and *D*. See Figure 10 for an example of *N_i_* variables. By $N=(N_{1},\ldots ,N_{l})$ and $M=(M_{1},\ldots ,M_{l})$ we denote the vectors of *N_i_* and *M_i_* variables, respectively.

Observe that $P[(ab,cd)]=P[i_{a}=i_{b},i_{c}=i_{d}]-P[(\mathrm{abcd})]$ and $P[(ac,bd)]=P[i_{a}=i_{c},i_{b}=i_{d}]-P[(\mathrm{abcd})]$. Further, note that when conditioned on specific values of **N** and **M**, *i_a_* = *i_c_* and *i_b_* = *i_d_* events become independent. That is, similarly to Claim 4.1, conditioning on the number of lineages at the divergence point for species *A* and *B* eliminates the dependency between *i_a_* and *i_b_* (and similarly for *i_c_* and *i_d_*). After conditioning on **N** and **M**, random variables $i_{a},i_{b},i_{c}$ and *i_d_* are all independent. In particular, we can think of lineages *a* and *b* as choosing one of the $\sum N_{i}$ lineages independently and uniformly at random. Similarly, *c* and *d* choose one of the $\sum M_{i}$ lineages independently and uniformly at random.

Then, for fixed values of the **N** and **M** vectors we have

$$\begin{array}{cc} P[i_{a}=i_{b},i_{c}=i_{d}|N,M] & =\sum_{j=1}^{l}(P[i_{a}=j|N]P[i_{b}=j|N])\\ & \;\;\;\cdot \sum_{j=1}^{l}(P[i_{c}=j|M]P[i_{d}=j|M])\\ & =\frac{\sum_{j}\left(N_{j}^{2}\right)}{{\left(\sum_{j}N_{j}\right)}^{2}}\frac{\sum_{j}\left(M_{j}^{2}\right)}{{\left(\sum_{j}M_{j}\right)}^{2}}. \end{array}$$

The last equality is due to $P[i_{a}=j|N]=\frac{N_{j}}{\sum_{i=1}^{l}N_{i}}$. That is, as mentioned above, due to the symmetry of the duplication/loss process *a* has a uniform probability of being ‘sampled’ from any of the lineages existing at the divergence point of species *A* and *B*. Similar relations can then be easily derived for *i_b_*, *i_c_* and *i_d_*.

Further, following the same idea, we have

$$P[i_{a}=i_{c},i_{b}=i_{d}|N,M]=\frac{\sum_{j}\left(N_{j}M_{j}\right)}{\left(\sum_{j}N_{j})(\sum_{j}M_{j}\right)}\frac{\sum_{j}\left(N_{j}M_{j}\right)}{\left(\sum_{j}N_{j})(\sum_{j}M_{j}\right)}.$$

Then, by Cauchy-Schwartz, ${\left(\sum_{j}\left(N_{j}M_{j}\right)\right)}^{2}\leq \sum_{j}\left(N_{j}^{2}\right)\sum_{j}\left(M_{j}^{2}\right)$ and therefore $P[i_{a}=i_{b},i_{c}=i_{d}|N,M]\geq P[i_{a}=i_{c},i_{b}=i_{d}|N,M]$ for any realization of vectors **N** and **M**. That is, P[(ab,cd)]≥P[(ac,bd)]. □

Using the above results, we have

P[ab|cd∈G,I]=P[(ab,cd)]≥P[(ac,bd)]=P[ac|bd∈G,I].

#### 4.1.3 Case I=(ab,c,d)∨(cd,a,b)∨(ac,b,d)∨(bd,a,c)

For convenience, from now on we denote the event (ab,c,d)∨(cd,a,b) by AB and the event (ac,b,d)∨(bd,a,c) by AC.

We prove that

$$\begin{array}{cc} & P[\mathrm{ab|cd}\in G,I]\\ & \;=P[\mathrm{ab|cd}\in G|AB]\;P[AB]+P[\mathrm{ab|cd}\in G|AC]\;P[AC]\\ & \;\geq P[\mathrm{ac|bd}\in G|AB]\;P[AB]+P[\mathrm{ac|bd}\in G|AC]\;P[AC]\\ & \;=P[\mathrm{ac|bd}\in G,I]. \end{array}$$

Consider the following results.

Lemma 4.4.
P[ab|cd∈G|AB]≥P[ac|bd∈G|AC] .

Proof. Note that fixing the number of root lineages allows us to treat the duplication/loss processes independently for the root edge and for the lower edges. Let $L_{r}$ be a duplication/loss scenario (i.e. a fixed realization of the duplication/loss process) in the root edge conditioned on *RL* = *l*. Then, without loss of generality assume that in case (*ab*, *c*, *d*), we have $i_{a}=i_{b}=1$, *i_c_* = 2 and *i_d_* = 3; in case (*cd*, *a*, *b*) we assume $i_{c}=i_{d}=1$, *i_a_* = 2 and *i_b_* = 3. Similarly, under (*ac*, *b*, *d*) we assume $i_{a}=i_{c}=1,i_{b}=2,i_{d}=3$ and under (*bd*, *a*, *c*) we assume that $i_{b}=i_{d}=1,i_{a}=2,i_{c}=3$. Then, a fixed $L_{r}$ scenario forces the same ‘top’ structure of the locus trees in all four cases.

Given that (*ab*, *c*, *d*) and (*cd*, *a*, *b*) cases are virtually identical for the remainder of the proof (since they are symmetric), for simplicity, we will only consider the (*ab*, *c*, *d*) case. Similarly, under the AC event, we will only consider case (*ac*, *b*, *d*).

**Fig. 11.**
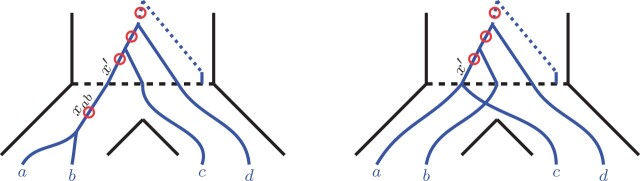
Caterpillar locus trees $L_{\left(ab,c,d\right)}$ (left) and $L_{\left(ac,b,d\right)}$ (right) embedded into the species tree. The red circles represent the potential duplication locations that could influence the gene tree probabilities. Note that the $L_{r}$ scenarios in the root edges are identical. That is, x′ lengths are equal, and the duplication locations above the dashed speciation lines are identical

**Fig. 12.**
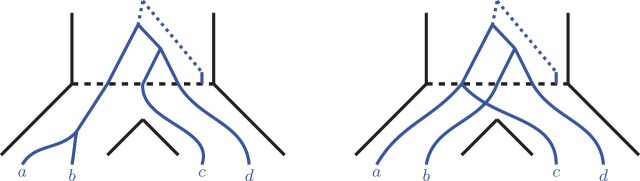
Balanced locus trees $L_{\left(ab,c,d\right)}$ (left) and $L_{\left(ac,b,d\right)}$ (right) embedded into the species tree

Then, Figures 11 and 12 depict two possible topologies of the $L_{r}$ scenario when acting on the root lineages 1, 2 and 3. Observe that the third topology, where root lineages 1 and 3 form a cherry, is identical in terms of analysis to the topology depicted in Figure 11, and therefore is not considered.

Note that in Figure 11, the resulting locus trees $L_{\left(ab,c,d\right)}$ and $L_{\left(ac,b,d\right)}$ are both caterpillars, while in Figure 12, the locus trees are both balanced. This separation is achieved because we condition on a fixed $L_{r}$ scenario. We now consider these two cases individually.

(i) $L_{\left(ab,c,d\right)}$ and $L_{\left(ac,b,d\right)}$ are caterpillars (see Fig. 11). Let *x_ab_* be the distance (in coalescent units) from the root speciation event to the divergence of *a* and *b* in the locus tree under the (*ab*, *c*, *d*) case (as shown on the figure). Note that $x_{ab}\geq 0$. There are two cases to consider.

*• There is a duplication along the x_ab_ lineage.* Then, as shown in Section 3.2.2, $P[\mathrm{ab|cd}\in G|AB,L_{r}]=1$. That is, $P[\mathrm{ab|cd}\in G|AB,L_{r}]\geq P[\mathrm{ac|bd}\in G|AC,L_{r}]$.

*• No duplications along the x_ab_ lineage.* Since $L_{\left(ab,c,d\right)}$ and $L_{\left(ac,b,d\right)}$ are both caterpillars, we denote their edges by *X* and *Y* as shown in Figure 6A. In particular we denote the *X* edge in $L_{\left(ab,c,d\right)}$ by $X_{\left(ab,c,d\right)}$ and the *X* edge in $L_{\left(ac,b,d\right)}$ by $X_{\left(ac,b,d\right)}$. Then, $w(X_{\left(ab,c,d\right)})=x\prime +x_{ab}$, whereas $w(X_{\left(ac,b,d\right)})=x\prime$ (note that x′ is as depicted in Fig. 11). Further, the two locus trees are identical in terms of the duplication locations in their internal edges.

Then, by Lemma 3.2, it is not difficult to see that $P[\mathrm{ab|cd}\in G|L_{r},(ab,c,d)]\geq P[\mathrm{ac|bd}\in G|L_{r},(ac,b,d)]$ for any fixed $L_{r}$. Therefore, the lemma holds.

(ii) $L_{\left(ab,c,d\right)}$ and $L_{\left(ac,b,d\right)}$ are balanced (see Fig. 12). By Lemma 3.1, $P[\mathrm{ab|cd}\in G|AB,L_{r}]=1$ and $P[\mathrm{ac|bd}\in G|AC,L_{r}]=1$. Note that we can apply Lemma 3.1, since the roots of the locus trees in these cases must be duplications.□

Lemma 4.5.
P[ab|cd∈G|AC]≥P[ac|bd∈G|AB] .

Proof. This result follows from Lemma 4.4 (i.e. P[ab|cd∈G|AB]≥P[ac|bd∈G|AC]) and the following relations:

$$\begin{array}{c} 2P[\mathrm{ab|cd}\in G|AC]+P[\mathrm{ac|bd}\in G|AC]=1;\\ 2P[\mathrm{ac|bd}\in G|AB]+P[\mathrm{ab|cd}\in G|AB]=1. \end{array}$$

□

Observation 4.3. *By Lemma 3.3, we have* P[ac|bd∈G|AC]≥P[ab|cd∈G|AC]*. Then, combining this with Lemma 4.5, we have* P[ac|bd∈G|AC]≥P[ac|bd∈G|AB].

Lemma 4.6. P[AB]≥P[AC].

Proof. We give the proof in Supplementary Section S2 of Supplementary Material. □

Summarizing the above results we have.

$$\begin{array}{cc} & P[\mathrm{ab|cd}\in G|AB]\;P[AB]+P[\mathrm{ab|cd}\in G|AC]\;P[AC]\\ & \geq P[\mathrm{ac|bd}\in G|AC]\;P[AB]+P[\mathrm{ac|bd}\in G|AB]\;P[AC]\\ & \geq P[\mathrm{ac|bd}\in G|AC]\;P[AC]+P[\mathrm{ac|bd}\in G|AB]\;P[AB]. \end{array}$$

Note that the first inequality is due to Lemmas 4.4 and 4.5. The last inequality is due to Lemma 4.6 and Claim 4.3.

That is, our main statement holds.

#### 4.1.4 Case I=(abc,d)∨(abd,c)∨(acd,b)∨(bcd,a)∨(abcd)

In all five cases locus tree *L* displays the quartet ab|cd. Therefore, by Lemma 3.3 P[ab|cd∈G|I]>P[ac|bd∈G|I]. Observe that we obtain the strict inequality in this case.

#### 4.1.5 Case I=(ad,bc)∨(ad,b,c)∨(bc,a,d)

In this case it is not difficult to see that *L* displays quartet ad|bc. Therefore (as can be seen from the derivations in Section 3), P[ab|cd∈G|I]=P[ac|bd∈G|I].

This concludes the proof for balanced *S*.

### 4.2 *S* is a caterpillar

**Fig. 13.**
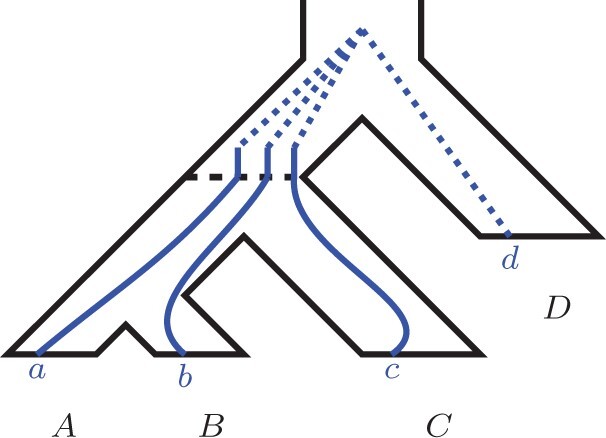
An example of the locus tree embedding into a caterpillar species tree. The three locus lineages crossing the dashed speciation line are the ABC-lineages

Without loss of generality assume that *S* is as it appears in Figure 13. Similarly to the balanced case, we implicitly condition the probability space on a fixed number of loci (lineages) existing at the moment of speciation as shown in the figure. Note that, while in the balanced case we considered root lineages, in the caterpillar scenario we consider lineages at the least common ancestor of *A*, *B* and *C*. That is, we refer to these lineages/loci as *ABC-lineages*. Finally, as in the balanced case, we denote the number of ABC-lineages by *l*.

We then use the $i_{a},i_{b},i_{c}$ notation in the same way as in the previous section (while referring to indices of ABC-lineages). Further, I={(a,b,c);(ab,c);(ac,b);(bc,a);(abc)} scenarios describe relations between *i_a_*, *i_b_* and *i_c_*.

We now prove that P[ab|cd∈G,I]≥P[ac|bd∈G,I] for all *I* in {(a,b,c);(ab,c)∨(ac,b);(bc,a);(abc)}. Moreover, for at least one such *I*, the strict inequality holds; in particular, see case 4.2.4 below.

#### 4.2.1 Case I=(a,b,c)

By the symmetry of the duplication/loss process, reshuffling the $i_{a},i_{b},i_{c}$ labels will not affect the probability of a fixed duplication/loss history in the root edge. Therefore, we have P[ab|cd∈G|I]=P[ac|bd∈G|I]=P[ad|bc∈G|I].

Then, P[ab|cd∈G,I]=P[ab|cd∈G|I] P[I]=P[ac|bd∈G|I] P[I]=P[ac|bd∈G,I].

#### 4.2.2 Case I=(ab,c)∨(ac,b)

The proof in this case is similar to case 4.1.3 for balanced *S*. In particular, observe the following.

Lemma 4.7. P[(ab,c)]≥P[(ac,b)].

Proof. It is sufficient to show that $P[i_{a}=i_{b}]\geq P[i_{a}=i_{c}]$. By Claim 4.2, $P[i_{a}=i_{c}]=1/l$. Further, Legried *et al.* (2020) showed that $P[i_{a}=i_{b}]\geq 1/l$ (see Lemma 1 in Legried *et al.* (2020)). □

Lemma 4.8.*The following holds.*

-
P[ab|cd∈G|(ab,c)]≥P[ac|bd∈G|(ac,b)] ;
-
P[ab|cd∈G|(ac,b)]≥P[ac|bd∈G|(ab,c)] ;
-
P[ac|bd∈G|(ac,b)]≥P[ac|bd∈G|(ab,c)] .

Proof. The proofs of these statements are similar to the proofs of the respective statements in Section 4.1.3. In particular, (i) corresponds to Lemma 4.4, (ii) corresponds to Lemma 4.5 and (iii) corresponds to Claim 4.3 from Section 4.1.3. □

Then, similarly to Section 4.1.3 we have

$$\begin{array}{cc} P[\mathrm{ab|cd}\in G,I] & =P[\mathrm{ab|cd}\in G|(ab,c)]\;P[(ab,c)]\\ & \;+P[\mathrm{ab|cd}\in G|(ac,b)]\;P[(ac,b)]\\ & \geq P[\mathrm{ac|bd}\in G|(ac,b)]\;P[(ab,c)]\\ & \;+P[\mathrm{ac|bd}\in G|(ab,c)]\;P[(ac,b)]\\ & \geq P[\mathrm{ac|bd}\in G|(ac,b)]\;P[(ac,b)]\\ & \;+P[\mathrm{ac|bd}\in G|(ab,c)]\;P[(ab,c)]\\ & =P[\mathrm{ac|bd}\in G,I]. \end{array}$$

#### 4.2.3 Case I=(bc,a)

In this case P[ab|cd∈G|I]=P[ac|bd∈G|I], since the locus tree displays the third quartet, ad|bc.

#### 4.2.4 Case I=(abc)

The locus tree displays quartet ab|cd; therefore, by Lemma 3.3 and the law of total probability, we have P[ab|cd∈G|I]>P[ac|bd∈G|I].

## 5 Consistency of ASTRAL-multi

We now extend our consistency result for ASTRAL-one to another variant of ASTRAL adapted to multi-locus input trees, called ASTRAL-multi.

Theorem 5.1. *Let* $S=(T_{S},w_{S})$  *be a fixed species tree and let* G  *be a collection of gene trees that independently evolved within S according to the DLCoal process. Then, as the number of trees in* G  *goes to infinity, the unrooted tree estimate by ASTRAL-multi converges almost surely to T_S_.*

Let *S* be a species tree with 4 leaves that displays AB|CD, and let *G* be a gene tree that evolved in *S* according to the DLCoal process. Let $G_{\mathrm{ab|cd}}$ (respectively $G_{\mathrm{ac|bd}}$ and $G_{\mathrm{ad|bc}}$) be the number of ab|cd (respectively ac|bd and ad|bc) quartets in *G*. Then, to prove Theorem 5.1 it is sufficient to show that the following result holds (Legried *et al.* (2020)):

Theorem 5.2. $E[G_{\mathrm{ab|cd}}]>\max\left(E[G_{\mathrm{ac|bd}}],E[G_{\mathrm{ad|bc}}]\right)$.

The remainder of the section is dedicated to the proof of Theorem 5.2. In fact, due to symmetry, it is sufficient to show that $E[G_{\mathrm{ab|cd}}]>E[G_{\mathrm{ac|bd}}]$. The general structure of the proof is similar to the proof of consistency for ASTRAL-one in the previous section. We present the proof for balanced *S*, and then briefly discuss the proof for caterpillar *S*.

Remark. *Some results in this section hold almost surely. Since this is sufficient for the proof of the theorem, we do not specify this explicitly.*

### 5.1 Proof of Theorem 5.2

As mentioned above, we assume that *S* is balanced. As before, we implicitly condition the probability space (and the expected values) on a fixed number of root lineages *l*. That is, we claim that Theorem 5.1 holds for any fixed value of *l*.

We now introduce our core notation for the proof. Similarly to the $G_{\mathrm{ab|cd}}$ notation, we let $L_{\mathrm{ab|cd}}$ (respectively $L_{\mathrm{ac|bd}}$ and $L_{\mathrm{ad|bc}}$) denote the number of ab|cd (respectively ac|bd and ad|bc) quartets in the locus tree *L*. Further, for a fixed scenario *I* (e.g. scenario $i_{a}=i_{b}=1,i_{c}=2,i_{d}=3$) let $L_{\mathrm{ab|cd}}^{I}$ be the number of ab|cd quartets in the locus tree that follow the scenario *I*. Further, $G_{\mathrm{ab|cd}}^{I}$ be the number of ab|cd quartets in *G* that ‘appeared’ from one of the $L_{\mathrm{ab|cd}}^{I}$ quartets. Similarly we define $L_{\mathrm{ac|bd}}^{I},L_{\mathrm{ad|bc}}^{I},G_{\mathrm{ac|bd}}^{I},$ and $G_{\mathrm{ad|bc}}^{I}$.

Consider any I≠(abcd). Note that the root of locus tree $L{|}_{\left\{a,b,c,d\right\}}$ must be a duplication for such *I* (because *I* involves at least two root lineages). Then, if *I* always defines balanced quartets, we have $G_{q}^{I}=L_{q}^{I}$ for any q∈{ab|cd,ac|bd,ad|bc} by Lemma 3.1. In particular, we note the following:

Observation 5.1. For any q∈{ab|cd,ac|bd,ad|bc} we have

$$\begin{array}{c} G_{q}^{\left(ab,cd\right)}=L_{q}^{\left(ab,cd\right)};\\ G_{q}^{\left(ac,bd\right)}=L_{q}^{\left(ac,bd\right)}. \end{array}$$

Further, we will only consider scenarios that uniquely determine the quartet types in the locus tree; therefore, we will typically omit the subscript in the $L_{q}^{I}$ notation. For example, we write $L^{\left(ab,cd\right)}$ instead of $L_{\mathrm{ab|cd}}^{\left(ab,cd\right)}$, since ab|cd is the only type of quartets that can appear under scenario (*ab*, *cd*).

Given a fixed root lineage *i*, let $A_{i}$ be the random variable denoting the number of *a* leaves generated by that lineage. Similarly, we define random variables $B_{i},C_{i},$ and $D_{i}$. By symmetry, $E[A_{1}]=E[A_{2}]=\cdots =E[A_{l}]$ (with similar relations holding for $B_{i},C_{i},D_{i}$). Then, observe the following:

Observation 5.2. Since the duplication/loss process runs independently in the parallel branches of the species tree, $X_{i}$ is independent from $Y_{j}$ for any X∈{A,B}, Y∈{C,D} and i,j∈{1,…,l}.

Observation 5.3. By the symmetry of the duplication/loss process, we have

$$E[X_{i}]=E[X_{j}]$$

*for all* X∈{A,B,C,D}  *and* i,j∈{1,2,…,l}.

Further, the following lemma is due to Legried *et al.*

Lemma 5.1 (Lemma 2 in Legried *et al.* (2020)).

$$\begin{array}{c} E[A_{1}B_{1}]\geq E[A_{1}]E[B_{1}];\\ E[C_{1}D_{1}]\geq E[C_{1}]E[D_{1}]. \end{array}$$

We now outline several key corollary statements.

Corollary 5.1.

$$\begin{array}{c} E[L^{\left(ab,cd\right)}]\geq E[L^{\left(ac,bd\right)}];\\ E[L^{\left(ab,c,d\right)}]\geq E[L^{\left(ac,b,d\right)}];\\ E[L^{i_{a}=i_{b}=1,\;i_{c}=2,\;i_{d}=3}]\geq E[L^{i_{a}=i_{c}=1,\;i_{b}=2,\;i_{d}=3}]. \end{array}$$

Proof. To prove the first relationship, note that the duplication/loss process occurs independently below distinct root lineages. Then, we have

$$\begin{array}{cc} E[L^{\left(ab,cd\right)}] & =l(l-1)E[A_{1}B_{1}]\;E[C_{1}D_{1}]\\ & \geq l(l-1)E[A_{1}]\;E[B_{1}]\;E[C_{1}]\;E[D_{1}]\\ & =l(l-1)E[A_{1}C_{1}]\;E[B_{1}D_{1}]=E[L^{\left(ac,bd\right)}]. \end{array}$$

The other two relationships can be established similarly. □

We now consider the following comprehensive set of scenarios: I∈{(a,b,c,d);(ab,cd)∨(ac,bd);(ab,c,d)∨(ac,b,d);(cd,a,b)∨(bd,a,c);(abc,d)∨(abd,c)∨(acd,b)∨(bcd,a)∨(abcd);(ad,bc)∨(ad,b,c)∨(bc,a,d)}. For each *I* we will prove that $E[G_{\mathrm{ab|cd}}^{I}]\geq E[G_{\mathrm{ac|bd}}^{I}]$ and for at least one *I* the strict inequality holds.

#### 5.1.1 Case I=(a,b,c,d)

By the symmetry of the duplication/loss process in the root edge, we have

$$E[G_{\mathrm{ab|cd}}^{\left(a,b,c,d\right)}]=E[G_{\mathrm{ac|bd}}^{\left(a,b,c,d\right)}]=E[G_{\mathrm{ad|bc}}^{\left(a,b,c,d\right)}].$$

#### 5.1.2 Case I=(ab,cd)∨(ac,bd)

By Claim 5.1, $G_{\mathrm{ab|cd}}^{I}=L^{\left(ab,cd\right)}$ and $G_{\mathrm{ac|bd}}^{I}=L^{\left(ac,bd\right)}$.

Then, combining this with Corollary 5.1, we have

$$E[G_{\mathrm{ab|cd}}^{I}]=E[L^{\left(ab,cd\right)}]\geq E[L^{\left(ac,bd\right)}]=E[G_{\mathrm{ac|bd}}^{I}].$$

#### 5.1.3 Case I=(ab,c,d)∨(ac,b,d)

Consider a fixed duplication-loss scenario, $L_{r}$, in the root edge of *S*. In this section we implicitly condition the probability space on $L_{r}$. That is, we prove that $E[G_{\mathrm{ab|cd}}^{I}]\geq E[G_{\mathrm{ac|bd}}^{I}]$ for each $L_{r}$.

Due to symmetry, we consider the following two core scenarios: $AB:=(i_{a}=i_{b}=1,i_{c}=2,i_{d}=3)$ and $AC:=(i_{a}=i_{c}=1,i_{b}=2,i_{d}=3)$. It is then sufficient to show the following:

Lemma 5.2.

$$E[G_{\mathrm{ab|cd}}^{AB}]+E[G_{\mathrm{ab|cd}}^{AC}]\geq E[G_{\mathrm{ac|bd}}^{AB}]+E[G_{\mathrm{ac|bd}}^{AC}].$$

Proof. Due to Lemma 4.4, it is not difficult to see that for any quartet on {a,b,c,d} that evolved according to scenario *AB* or *AC*, we have P[ab|cd∈G|AB]≥P[ac|bd∈G|AC]. Therefore, we have

$$E[G_{\mathrm{ab|cd}}^{AB}]\geq E[L^{AB}]\;P[\mathrm{ac|bd}\in G|AC].$$

Similarly, due to Lemma 4.5, we know that P[ab|cd∈G|AC]≥P[ac|bd∈G|AB]. Therefore,

$$E[G_{\mathrm{ac|bd}}^{AB}]\leq E[L^{AB}]\;P[\mathrm{ab|cd}\in G|AC].$$

Further, note that P[ac|bd∈G|AC] and P[ab|cd∈G|AC] do not depend on the choice of the {a,b,c,d} lineages, but only depend on the scenario $L_{r}$ (see Figs 11 and 12 (right)). Hence,

$$\begin{array}{c} E[G_{\mathrm{ac|bd}}^{AC}]=E[L^{AC}]\;P[\mathrm{ac|bd}\in G|AC];\\ E[G_{\mathrm{ab|cd}}^{AC}]=E[L^{AC}]\;P[\mathrm{ab|cd}\in G|AC] \end{array}$$

Combining all of the above relations we have

$$\begin{array}{c} \begin{array}{cc} E[G_{\mathrm{ab|cd}}^{AB}]+E[G_{\mathrm{ab|cd}}^{AC}]\geq & E[L^{AB}]\;P[\mathrm{ac|bd}\in G|AC]\\ & +E[L^{AC}]\;P[\mathrm{ab|cd}\in G|AC]; \end{array}\\ \begin{array}{cc} E[G_{\mathrm{ac|bd}}^{AC}]+E[G_{\mathrm{ac|bd}}^{AB}]\leq & E[L^{AC}]\;P[\mathrm{ac|bd}\in G|AC]\\ & +E[L^{AB}]\;P[\mathrm{ab|cd}\in G|AC]. \end{array} \end{array}$$

We can now conclude the proof by noting that $E[L^{AB}]\geq E[L^{AC}]$ (by Corollary 5.1) and P[ac|bd∈G|AC]>P[ab|cd∈G|AC] (by Lemma 3.3). □

#### 5.1.4 Case I=(cd,a,b)∨(bd,a,c)

This case is symmetric to I=(ab,c,d)∨(ac,b,d). Therefore, the proof is similar.

#### 5.1.5 Case I=(abc,d)∨(abd,c)∨(acd,b)∨(bcd,a)∨(abcd)

All quartets in the locus tree under each of these scenarios are ab|cd. Then, by Lemma 3.3, P[ab|cd∈G|I]>P[ac|bd∈G|I] for each of the $L_{\mathrm{ab|cd}}^{I}$ quartets. Therefore,

$$E[G_{\mathrm{ab|cd}}^{I}]>E[G_{\mathrm{ac|bd}}^{I}].$$

#### 5.1.6 Case I=(ad,bc)∨(ad,b,c)∨(bc,a,d)

All quartets in the locus tree under each of these scenarios are ad|bc. It is then not difficult to see that $E[G_{\mathrm{ab|cd}}^{I}]=E[G_{\mathrm{ac|bd}}^{I}]$.

### 5.2 Caterpillar species tree

We now briefly discuss the proof strategy for Theorem 5.2 when *S* is a caterpillar. Similarly to Section 4.2, we condition the duplication/loss process on a fixed number of ABC-lineages (*l*)—see Figure 13. Adapting a similar notation to Section 5.1, let $A_{i},B_{i},C_{i}$ denote the random variables for the number of *a*, *b* and *c* genes, respectively, below the *i*th ABC-lineage (in the locus tree). Further, let D denote the total number of *d* leaves. It is then not difficult to show that D is independent from $X_{i}$ for any X∈{A,B,C}. Further, $A_{i}$ and $B_{i}$ are independent from $C_{j}$ for any i,j∈{1,2,…,l} (analogously to Claim 5.2). Claim 5.3 also upholds when we restrict X to {A,B,C}. Finally, Lemma 5.1 is applicable in the caterpillar case as well; i.e. $E[A_{1}B_{1}]\geq E[A_{1}]E[B_{1}]$.

We now need to consider the following scenarios: I∈{(a,b,c);(ab,c)∨(ac,b);(bc,a);(abc)} and prove that $G_{\mathrm{ab|cd}}^{I}\geq G_{\mathrm{ac|bd}}^{I}$ for all such *I*. It is then not difficult to do so, since I=(a,b,c) is analogous to Case 5.1.1 from Section 5.1, I=(ab,c)∨(ac,b) is analogous to Case 5.1.3, I=(bc,a) is analogous to Case 5.1.6, and I=(abc) is analogous to Case 5.1.5. Further, under I=(abc) the inequality $G_{\mathrm{ab|cd}}^{I}>G_{\mathrm{ac|bd}}^{I}$ is strict, similarly to Case 5.1.5. That is, Theorem 5.2 holds.

## 6 Conclusion

For the first time, we investigated and established statistical properties of a popular species tree inference method under the powerful duplication-loss-coalescence model. We proved that two natural versions of ASTRAL (adapted for the duplication-loss shaped gene families) are statistically consistent under DLCoal. Our result reinforces the practical value of ASTRAL and other quartet-based methods in the area of evolutionary inference. In addition to our work, Hill *et al.* (2020) studied the rate of convergence of ASTRAL under DLCoal. In the future, we anticipate that other statistically consistent methods under DLCoal will be discovered, and the methods will be compared based on their theoretical rate of convergence and simulation studies, advancing the accuracy of evolutionary inference.

*Financial Support*: This material is based upon work supported by the National Science Foundation under Grant No. 1617626. The Department of Defense, Defense Advanced Research Projects Agency, Preventing Emerging Pathogenic Threats program (HR00112020034 to OE). During the revision and editing, AM was funded by the USDA Agricultural Research Service Research Participation Program of the Oak Ridge Institute for Science and Education (ORISE) through an interagency agreement between the U.S. Department of Energy (DOE) and USDA Agricultural Research Service (contract number DE-AC05-06OR23100). Mention of trade names or commercial products in this article is solely for the purpose of providing specific information and does not imply recommendation or endorsement by the USDA, DOE or ORISE. USDA is an equal opportunity provider and employer.

*Conflict of Interest*: The authors declare that they have no conflict of interest.

## Supplementary Material

btab414_Supplementary_Data
