# Supplementary material for: Quartet-based inference is statistically consistent under the unified duplication-loss-coalescence model
Source: Bioinformatics. 2021 May 28;37(22):4064–74. doi: 10.1093/bioinformatics/btab414 (PMC9113308; doi:10.1093/bioinformatics/btab414)
Supplement: btab414_Supplementary_Data [file btab414_supplementary_data.pdf]

Quartet-Based Inference is Statistically Consistent Under the Unified Duplication-Loss-Coalescence Model

Alexey Markin
Virus and Prion Research Unit
National Animal Disease Center, USDA-ARS
alexey.markin@usda.gov

Oliver Eulenstein
Department of Computer Science
Iowa State University
oeulenst@iastate.edu

S1 Proofs of Lemmas 3.2 and 3.3

In this section we use t to denote the edge length from the lowest duplication point (on the internal edges of L) to the first vertex below it (as in Sections 3.1.3, 3.2.3, and 3.2.4).

Proof of **Lemma 3.2**. The statement is trivial for cases from Sections 3.2.1 (no duplications) and 3.2.2 (X-edge duplication). Therefore, we now prove the statement for cases from Sections 3.2.3 and 3.2.4.

- *Case 3.2.3* (Y-edge duplication). It is sufficient to show that $P[a, b, c$ coalesced before duplication$]$ grows as $x := \omega(X)$ grows. Consider the following relation:

  $$P[a, b, c \text{ coalesced before duplication}] = g_{2,1}(x)g_{2,1}(t) + g_{2,2}(x)g_{3,1}(t).$$

  Observe that for any $x_1 > x_2$ we have $g_{2,1}(x_1) = 1 - e^{-x_1} > 1 - e^{-x_2} = g_{2,1}(x_2)$. Then we also have

  $$g_{2,1}(x_1)g_{2,1}(t) + g_{2,2}(x_1)g_{3,1}(t) > g_{2,1}(x_2)g_{2,1}(t) + g_{2,2}(x_2)g_{3,1}(t),$$

  since $g_{2,1}(t) > g_{3,1}(t)$ and $g_{2,1}(x) + g_{2,2}(x) = 1$ for all positive x and t.

- *Case 3.2.4* (root-edge duplication). In this case it is sufficient to prove that $P[a, b, c, d$ coalesced before the duplication$]$ grows as x grows. Observe that

  $$\begin{aligned} &P[a, b, c, d \text{ coalesced before the duplication}] \\ &= g_{2,1}(x)P[3 \text{ lineages coalesced on Y and root edges before the duplication}] \\ &\quad + g_{2,2}(x)P[4 \text{ lineages coalesced on Y and root edges before the duplication}] \\ &= g_{2,1}(x) \cdot P_3 + g_{2,2}(x) \cdot P_4. \end{aligned}$$

  We introduced constants P_3 and P_4 above to simplify the notation.

  It is now left to show that $P_3 > P_4$ with the remainder of the proof following similarly to Case 3.2.3 above.

  Note that

  $$P_3 = g_{2,1}(y)g_{2,1}(t) + g_{2,2}(y)g_{3,1}(t)$$

  and

  $$P_4 = g_{3,1}(y)g_{2,1}(t) + g_{3,2}(y)g_{3,1}(t) + g_{3,3}(y)g_{4,1}(t).$$

Then

$$\begin{aligned} P_3 - P_4 &= g_{2,1}(t)\big(g_{2,1}(y) - g_{3,1}(y)\big) + g_{3,1}(t)\big(g_{2,2}(y) - g_{3,2}(y)\big) - g_{4,1}(t)g_{3,3}(y) \\ &> g_{3,1}(t)\big(g_{2,1}(y) - g_{3,1}(y) + g_{2,2}(y) - g_{3,2}(y)\big) - g_{4,1}(t)g_{3,3}(t) \\ &= g_{3,1}(t)\big(1 - g_{3,1}(y) - g_{3,2}(y)\big) - g_{4,1}(t)g_{3,3}(y) \\ &> g_{4,1}(t)\big(1 - g_{3,1}(y) - g_{3,2}(y) - g_{3,3}(y)\big) = g_{4,1}(t)(1-1) = 0. \end{aligned}$$

The above inequalities hold due to $g_{2,1}(z) > g_{3,1}(z) > g_{4,1}(z)$ for any positive z.

□

Proof of **Lemma 3.3**. Observe that this statement is not trivial only in the following three cases:

(i) L is balanced, and the lowest duplication is at the root edge (Case 3.1.3). To prove that $P[\mathsf{ab|cd} \in G \mid L] > P[\mathsf{ac|bd} \in G \mid L]$ it is sufficient to show that

$$P[a,b,c,d \text{ coalesced before duplication}] \geq g_{4,1}(t).$$

Observe then that

$$P[a,b,c,d \text{ coalesced before duplication}] = \sum_{k=2}^{4} g_{k,1}(t)P[k \text{ lineages entered the root edge}] \geq g_{4,1}(t).$$

The inequality holds since $g_{k,1}(t) \geq g_{4,1}(t)$ for all $k \in \{2,3,4\}$ and

$$\sum_{k=2}^{4} P[k \text{ lineages entered the root edge}] = 1.$$

(ii) L is a caterpillar, and the lowest duplication is on the Y edge (Case 3.2.3). In this case, it is sufficient to show that $P[a,b,c \text{ coalesced before duplication}] \geq g_{3,1}(t)$. Similarly to the above case, observe that

$$P[a,b,c \text{ coalesced before duplication}] = \sum_{k=2}^{3} g_{k,1}(t)P[k \text{ lineages entered edge } Y] \geq g_{3,1}(t).$$

Then the inequality holds since $g_{k,1}(t) \geq g_{3,1}(t)$ for all $k \in \{2,3\}$.

(iii) L is a caterpillar, and the lowest duplication is at the root edge (Case 3.2.4). In this case, we need to show that

$$P[a,b,c,d \text{ coalesced before the duplication}] \geq g_{3,2}(y)g_{3,1}(t) + g_{3,1}(y)g_{2,1}(t) + g_{3,3}(y)g_{4,1}(t).$$

Consider now the following relation that comes from the proof of Lemma 3.2:

$$\begin{aligned} &P[a,b,c,d \text{ coalesced before the duplication}] \\ &= g_{2,1}(x)P[\text{3 lineages coalesced on Y and root edges before the duplication}] \\ &\quad + g_{2,2}(x)P[\text{4 lineages coalesced on Y and root edges before the duplication}] \\ &= g_{2,1}(x) \cdot P_3 + g_{2,2}(x) \cdot P_4. \end{aligned}$$

Then, using the P_3 and P_4 notation, we need to show that

$$g_{2,1}(x)P_3 + g_{2,2}(x)P_4 \geq P_4.$$

Note that $g_{2,1}(x) + g_{2,2}(x) = 1$. Further, in the proof of Lemma 3.2 we show that $P_3 \geq P_4$. Then the inequality follows.

□

S2 Proof of Lemma 4.6

Proof. First of all, note that for, e.g., the (ab, c, d) case to be feasible, we need to have at least three root lineages. That is, $l \geq 3$. Next, observe that

$$\begin{aligned} P[AB] = P[(ab, c, d) \vee (cd, a, b)] &= P[i_a = i_b, i_c \neq i_d] - P[(abc, d)] - P[(abd, c)] \\ &+ P[i_c = i_d, i_a \neq i_b] - P[(acd, b)] - P[(bcd, a)]; \\ P[AC] = P[(ac, b, d) \vee (bd, a, c)] &= P[i_a = i_c, i_b \neq i_d] - P[(abc, d)] - P[(acd, b)] \\ &+ P[i_b = i_d, i_a \neq i_c] - P[(abd, c)] - P[(bcd, a)]. \end{aligned}$$

Therefore, it is sufficient to show that

$$P[i_a = i_b, i_c \neq i_d] + P[i_c = i_d, i_a \neq i_b] \geq P[i_a = i_c, i_b \neq i_d] + P[i_b = i_d, i_a \neq i_c].$$

Let $x := P[i_a = i_b]$ and $y := P[i_c = i_d]$. Recall that, by Lemma 4.1, $x, y \geq \frac{1}{l}$. Then,

$$P[i_a = i_b, i_c \neq i_d] + P[i_c = i_d, i_a \neq i_b] = x(1-y) + y(1-x) = x + y - 2xy. \quad (1)$$

Further,

$$\begin{aligned} P[i_b = i_d \mid i_a = i_c] &= \sum_{j=1}^{l} P[i_b = i_d \mid i_a = i_c = j] P[i_a = i_c = j \mid i_a = i_c] && (2) \\ &= \frac{1}{l} \sum_{j=1}^{l} \sum_{k=1}^{l} P[i_b = i_d = k \mid i_a = i_c = j] && (3) \\ &= \frac{1}{l} \sum_{j=1}^{l} \sum_{k=1}^{l} P[i_b = k \mid i_a = j] P[i_d = k \mid i_c = j] && (4) \\ &= \frac{1}{l} l \Big(P[i_b = 1 \mid i_a = 1] P[i_d = 1 \mid i_c = 1] + \ldots + P[i_b = l \mid i_a = 1] P[i_d = l \mid i_c = 1] \Big) && (5) \\ &= xy + (l-1) \frac{(1-x)}{(l-1)} \frac{(1-y)}{(l-1)}. && (6) \end{aligned}$$

- The transition to equality 3 is due to $P[i_a = i_c = j \mid i_a = i_c] = \frac{P[i_a = i_c = j]}{P[i_a = i_c]} = \frac{1/l^2}{1/l} = 1/l$ (via Claims 4.1 and 4.2).
- The transition to equality 4 is due to the independence of the i_b and i_d random variables (as well as of i_a and i_c).
- To understand the transition to equalities 5 and 6, observe that (due to the symmetry of the duplication/loss process)

  $$P[i_b = k \mid i_a = k] = P[i_b = 1 \mid i_a = 1] = x$$

  for any k, and

  $$P[i_b = k \mid i_a = j] = P[i_b = k \mid i_a = 1] = \frac{1-x}{l-1}$$

  for any $k \neq 1$ and $j \neq k$.

  Similarly, we have

  $$P[i_d = k \mid i_c = k] = P[i_d = 1 \mid i_c = 1] = y$$

for any k, and

$$P[i_b = k \mid i_a = j] = P[i_b = k \mid i_a = 1] = \frac{1-y}{l-1}$$

for any $k \neq 1$ and $j \neq k$.

Then,

$$P[i_a = i_c, i_b \neq i_d] = (1 - P[i_b = i_d \mid i_a = i_c])P[i_a = i_c] = (1 - xy - \frac{(1-x)(1-y)}{(l-1)})\frac{1}{l};$$

$$P[i_a = i_c, i_b \neq i_d] + P[i_b = i_d, i_a \neq i_c] = \frac{2}{l(l-1)}(l - 2 - lxy + x + y). \quad (7)$$

Multiplying Equations 1 and 7 by $l(l-1)$ and fixing some $y \in [1/l, 1]$ we obtain two **linear** functions.

$$f(x) := l(l-1)(x + y - 2xy)$$
$$g(x) := 2(l - 2 - lxy + x + y).$$

It is then sufficient to show that $f(1/l) \geq g(1/l)$ and $f(1) \geq g(1)$ to conclude the proof (since x is in the $[1/l, 1]$ range).

$$f(1/l) = l - 1 + y(l-1)(l-2);$$
$$g(1/l) = 2l - 4 - 2y + 2/l + 2y = 2l - 4 + 2/l.$$

Observe that $f(1/l)$ is minimum when $y = 1/l$ (since that is the smallest possible value for y). In that case $f(1/l) = l - 1 + (l^2 - 3l + 2)/l = 2l - 4 + 2/l$. That is, $f(1/l) \geq g(1/l)$ for all values of y. Let us now compare $f(1)$ and $g(1)$.

$$f(1) = l(l-1)(1-y);$$
$$g(1) = 2(l - 2 - ly + 1 + y) = 2(l - 1 - y(l-1)) = 2(l-1)(1-y).$$

It is then not difficult to see that $f(1) \geq g(1)$ for all $l \geq 3$. $\square$
